# The putty kidney: a classic sign from past in genitourinary radiology

**DOI:** 10.11604/pamj.2022.42.16.34128

**Published:** 2022-05-09

**Authors:** Savas Deftereos, Soultana Foutzitzi

**Affiliations:** 1 Radiology Department, Democritus University of Thrace, Alexandroupolis, Greece

**Keywords:** Putty kidney, tuberculosis, autonephrectomy, computed tomography

## Image in medicine

A 56-year-old woman with known genitourinary tuberculosis (GUTB) presented for follow-up evaluation with Computed Tomography (CT). Her urine referred acidic with few pus cells, but sterile on culture. Other laboratory tests were normal. A pigtail catheter is also referred to the left kidney. X-ray (actually CT-topogram) kidney, ureter and bladder (KUB) show multiple lobulated calcifications in the right renal region conglomerated into the shape of the kidney. This imaging finding were suggestive of the classical “putty kidney sign” of an end stage renal tuberculosis (TB). Radiological findings in renal TB depend on the extent of the disease process and are best demonstrated both on intravenous urogram and CT scan. Describing the condition, it is an extensive parenchymal calcification in a non-functioning kidney, forming its cast. The “putty kidney” or “autonephrectomy” is the end result of scarring, obstruction, and atrophy of the kidney due to TB which is complicated by dystrophic calcification with time. Tuberculosis of the kidney results from hematogenous seeding of M tuberculosis in the glomerular and peritubular capillary bed from primary pulmonary infection. Disease´s physiopathology and/or progression includes granuloma formation which causes necrosis with cavitation within the renal parenchyma, fibrosis, calcium deposition and stricture formation. Diagnosis of GUTB based on biochemical workup is often delayed and high index of suspicion required to avoid misdiagnosis. Thus, imaging has an important role in diagnosis. The present X-ray KUB and CT is a classic example of such “putty kidney” which first described in 1906 by Dr. F. Tilden Brown, a genitourinary surgeon.

**Figure 1:**
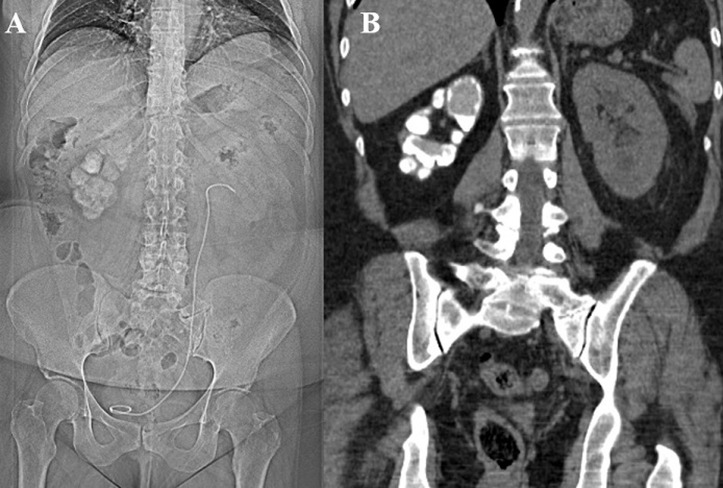
(A) CT scout view (topogram, instead of X-ray kidney, ureter and bladder) revealing diffuse alveolar calcification with kidney shape in right renal region and a well-positioned pigtail catheter; (B) computed tomography image showing the densely calcified right kidney

